# Supplementary material for: Association of hypoglycemic events with cognitive impairment in patients with type 2 diabetes mellitus: Protocol for a dose-response meta-analysis
Source: PLoS One. 2024 Feb 2;19(2):e0296662. doi: 10.1371/journal.pone.0296662 (PMC10836671; doi:10.1371/journal.pone.0296662)
Supplement: S2 Appendix — (DOCX) [file pone.0296662.s002.docx]

**Search strategy**

**Take PubMed as an example**

**#1 ((Diabetes Mellitus,Type 2[MeSH Terms]) OR (noninsulin[Title/Abstract])) OR (noninsulin-dependent Diabetes Mellitus[Title/Abstract])) OR (Type 2 diabetes[Title/Abstract])) OR (Type 2 diabetes Mellitus[Title/Abstract])) OR (Type 2 diabetic[Title/Abstract])) OR (T2DM[Title/Abstract])) OR (DM[Title/Abstract]))**

**#2 (((Cognitive Dysfunction[MeSH Terms]) OR (Cognition Disorders[Title/Abstract])) OR (Cognitive Disorder[Title/Abstract])) OR (Dementia[Title/Abstract])) OR (cognitive decline[Title/Abstract])) OR (cognition disorder[Title/Abstract])) OR (cognition disorder[Title/Abstract])) OR (cognitive impairment[Title/Abstract])) OR (executive function[Title/Abstract])) OR (cognitive function[Title/Abstract])) OR (neurodegeneration[Title/Abstract])) OR (neurodegenerative disease[Title/Abstract])) OR (neurocognitive disorder[Title/Abstract])) OR (neuropsychiatric disorder[Title/Abstract])) OR (mental deterioration[Title/Abstract])) OR (dysmnesia[Title/Abstract]))OR (learning-memorizing ability[Title/Abstract])) OR (allomnesia[Title/Abstract])) OR (memory[Title/Abstract])))**

**#3 ((Risk factor[Title/Abstract]) OR (predicted[Title/Abstract])) OR (predictor[Title/Abstract])) OR (risk[Title/Abstract])) OR (relat[Title/Abstract])) OR (associat[Title/Abstract])) OR (factor[Title/Abstract])) OR (reason[Title/Abstract])) OR (correlated[Title/Abstract])) OR (predictor[Title/Abstract])) OR (relevan[Title/Abstract])) OR (influen[Title/Abstract])) OR (inciden[Title/Abstract]))**

**#4 #1 AND #2 AND #3**
