# Supplementary material for: Association of hypoglycemic events with cognitive impairment in patients with type 2 diabetes mellitus: Protocol for a dose-response meta-analysis
Source: PLoS One. 2024 Feb 2;19(2):e0296662. doi: 10.1371/journal.pone.0296662 (PMC10836671; doi:10.1371/journal.pone.0296662)
Supplement: Attachment — Submitted filename: Response to Reviewers.docx [file pone.0296662.s005.docx]

**Response to Reviewers “Association of hypoglycemic events with cognitive impairment in patients with type 2 diabetes mellitus: Protocol for a dose-response meta-analysis”**

**Min Ye, Ai Hong Yuan, Qi Qi Yang, Qun Wei Li, Fei Yue Li, Yan Wei**

**Date	November 2023**

Dear Editor,

We would like to express our sincere thanks for giving us the valuable opportunity to revise our manuscript. Meanwhile, thanks to four reviewers for their constructive comments.

The revised version of our manuscript has been modified based on their helpful comments. All the revisions to the manuscript are marked in red font. In the following correspondence, we describe the changes made in this version. We hope that now the paper is ready for publication.

Looking forward to hearing your response soon.

Thank you and best regards.

Yours sincerely,

Min Ye

Corresponding author:

Name: Ai Hong Yuan

E-mail: 490603279@qq.com; yuanah2023@163.com.

**Response to journal's requirements**

**1. Please ensure that your manuscript meets PLOS ONE's style requirements, including those for file naming.**

**Ans**: We appreciate you reminding us. We have made the necessary formatting adjustments in accordance with PLOS ONE style guidelines, and we trust that the manuscript now satisfies your criteria.

**2.We note that the grant information you provided in the ‘Funding Information’ and ‘Financial Disclosure’ sections do not match. When you resubmit, please ensure that you provide the correct grant numbers for the awards you received for your study in the ‘Funding Information’ section.**

**Ans**: We appreciate you reminding us. We have reviewed the funding information portion of the manuscript and determined that the data it contains is correct. We apologize for any trouble this has given you with your work and will be updating the incorrect information in the online submission form.

**3.Your ethics statement should only appear in the Methods section of your manuscript. If your ethics statement is written in any section besides the Methods, please move it to the Methods section and delete it from any other section. Please ensure that your ethics statement is included in your manuscript, as the ethics statement entered into the online submission form will not be published alongside your manuscript.**

**Ans**: We appreciate your proposal, and as per it, we have relocated the ethical statement to the manuscript methodology section and deleted it from its previous location.

**Response to Reviewer 1's Comments**

Firstly, we would like to express our sincere thanks for your constructive comments and positive response. The revised version of the manuscript has been significantly modified based on your comments. The revised parts are marked in red font. We hope you are satisfied with our revisions**.**

**1. You can further expand the keywords used for the literature search. (For example: neurodegeneration, neurodegenerative disease, neurocognitive disorder, neuropsychiatric disorder) This will help researchers screen for all relevant studies.**

**Ans**: We have broadened relevant search terms based on your suggestions to completely choose relevant papers, as shown in lines 100-115 and S2 Appendix.

**2.** **You can make the information used for data extraction more detailed. Definition of cognitive impairment, Cognitive impairment measurements, etc.**

**Ans:** Thanks to your advice, we have modified the required data extraction information, making our scheme more complete and practicable, as shown in lines 148-160.

**3. You can define the variables to be used for dose-response analysis in more detail.**

**Ans:** We specified possible variables for dose-response analysis based on your suggestions, such as frequency of hypoglycemia, severity of hypoglycemia, duration of hypoglycemia, and incidence of cognitive impairment, to provide a clearer picture of our study protocol. For more information, see lines 154-160 and 197-202.

**Response to Reviewer 2's Comments**

Firstly, we would like to express our sincere thanks for your constructive comments and positive response. The revised version of the manuscript has been significantly modified based on your comments. The revised parts are marked in red font. We hope you are satisfied with our revisions.

**1.Eligibility criteria. Detail inclusion criteria for exposure, such as** **definition for** **hypoglycemic events or assessment methods. Furthermore, specify criteria for outcome (****cognitive impairment), i.e., assessment methods, definition, et**c**.**

**Ans**: Thanks for your valuable advice, we have added the assessment methods for hypoglycemic events and cognitive impairment in the inclusion criteria section according to your suggestion. See lines 123-131 for details.

**2. Line 96. Specify the type of observational studies to be included.**

**Ans**: We have clarified the study types of the included observational research, such as cross-sectional studies, case-control studies, cohort studies, and so on, in response to your recommendation. For more information, see lines 118-119 of the text.

**3.Line 97. “Individuals with a diagnosis of T2DM will be incorporated”. Will only studies that report medical diagnosis be included or could they be self-reported? Please specify. Also, will the participants included be adults? Please specify and indicate age range.**

**Ans**: Thank you for your extremely useful advice. We clarified the characteristics of subjects in related studies based on your ideas. To assure the scientific validity and rigor of the trial results, we demand that all subjects be medically diagnosed with type 2 diabetes and be adults, that is, above the age of 18. Details can be found in lines 120-121.

**4.Line 103. Please delete criterion 1. This is a repeated criterion that has already been established as an inclusion criterion**.

**Ans**: Thank you for your wise counsel. This is the result of our carelessness. We removed criterion 1 based on your advice.

**5.** **Line 153. Will a minimum number of studies be considered for meta-analysis? Will different meta-analyses be performed depending on the study design? Please detail.**

**Ans**: Thank you for your valuable advice and in accordance with the Cochrane Handbook for Systematic Reviews, we have stipulated that meta-analyses will be performed only if there are sufficient studies (≥2) with similar definitions of exposure and outcome of interest, and only descriptive analyses will be performed if the number of studies is insufficient. When the number of studies and relevant data are sufficiently rich, we will conduct subgroup analysis according to different study types, such as cross-sectional studies, case-control studies, cohort studies, etc. See lines 176-177 and 190 of the text for details.

**6. Line 157. Provide the interpretation of I^2^ according to the percentages of heterogeneity and its respective reference.**

**Ans**：We have updated the explanation of I^2^ for the proportion of heterogeneity and its respective reference, as you requested. Details can be found on lines 181-186.

**7.** **Line 165. Will a minimum of studies be considered for meta-regression analyses? Consider Cochrane recommendations.**

**Ans**: Thank you for your insightful advice. According to the Cochrane Handbook for Systematic Reviews of Interventions, the minimum number of studies for meta-regression analysis is ten. Look at lines 192-193.

**8. For subgroup and meta-regression analyses consider** **BMI status and** **physical activity levels. Also, add these variables for data extraction.**

**Ans**: Thank you for your helpful suggestions. We completely agree with your points of view and will include BMI status and physical activity levels in the data extraction, subgroup analysis, and meta-regression, making our study more valuable. For more information, see lines 152 and 189.

**9. Lines 171-172. Please provide more detail on the possible dose-response curve analysis to be performed.**

**Ans**: We have added possible exposure factors and outcome measures that may be utilized for dose-response analyses to the dose-response curve analysis section based on your recommendation. See lines 197-202 for more information.

**10.** **Lines 174-175. Will a minimum of studies be considered for publication bias? Consider Sterne et al. (2011) recommendations. Moreover, for the linear regression approach, what will be the p-value considered significant?**

**Ans**: Thank you for your advice. We concluded that the minimal number of literature with publication bias is ten based on your proposal and relevant literature. At the same time, a P value of less than 0.05 for Egger's regression asymmetry test is considered severe publication bias. For more information, see lines 203-206.

**11.** **Lines 177-178. Specify the characteristics of the levels of evidence. Detail the levels of evidence possible to be graded according to GRADE. What does high, medium, low and very low mean?**

**Ans**: We appreciate your recommendations, which helped us clarify the characteristics of the evidence level and further explain the GRADE evidence level. For more information, see lines 207-212.

**12. I think it would be useful for the authors to discuss the potential role of exercise, sleep, and other lifestyle behaviors in the association between hypoglycemic events and cognitive decline in patients with type 2 diabetes mellitus. In this sense, manuscripts with an integrative and translational character should be included in the discussion, such as doi:** **https://doi.org/10.1186/s12902-019-0402-3; doi: https://doi.org/10.1586/eem.10.78; doi:** **https://doi.org/10.4093/dmj.2022.0007; doi:** **https://doi.org/10.1136/bmj-2022-072691; and doi: http://dx.doi.org/10.1136/bjsports-2022-106355**

**Ans**: Thank you for your detailed constructing proposal. We read the appropriate literature thoroughly and gained greatly. Lifestyle influences the link between hypoglycemic occurrences and cognitive impairment, which has crucial implications for future studies in this area. We have added relevant content to the discussion section based on your recommendation. Details can be found in lines 245-261.

**Response to Reviewer 3's Comments**

Firstly, we would like to express our sincere thanks for your constructive comments and positive response. The revised version of the manuscript has been significantly modified based on your comments. The revised parts are marked in red font. We hope you are satisfied with our revisions.

**1.“The connection that exists between hypoglycemic occurrences and the likelihood of cognitive impairment will be investigated through an organized review of multiple databases.” It is better to include a sentence summarising the inclusion criteria: "Studies that ........ were included".**

**Ans**: Thank you for your proposal; we have provided the summary discourse of the literature inclusion criteria based on it. See lines 25-26.

**2. “Both Chinese and English literature…” Remove this from the summary.**

**Ans**: Thank you for your important advice; we have removed "Both Chinese and English literature..." following your recommendation.

**3. “…normal individuals” Change from normal to healthy**

**Ans**: We modified "normal" to "healthy" based on your advice. See line 56 for further information.

**4. “By constructing a diabetic rat model, Seok Joon Won…”. Better: "In animal models, it has been observed that..."**

**Ans**: Thank you for your helpful advice. We changed the sentence structure based on your ideas. For more information, see line 67.

**5. “This investigation seeks to update the systematic review and meta-analysis by including the latest relevant studies”. Better “This systematic review and meta-analyses aimed to…”**

**Ans**: Thank you for your valuable suggestions. We have revised the sentence structure according to your suggestions. See lines 85-89 for details.

**6. If it is a meta-analysis of observational studies, the MOOSE guideline should also be included.**

**Ans**: Thank you for your valuable suggestions, and as indicated in the methodology section, we will report our work in compliance with the MOOSE guidelines. Details can be found on lines 95-97.

**7.“All observational studies published in Chinese or English will be included”. Why not include other languages? Even if authors only know English and Chinese, there are translation tools that allow studies in other languages to be included.**

**Ans**: Thank you for your proposal; we studied it carefully and ultimately chose to incorporate as many literatures in diverse languages as feasible, resulting in more scientifically robust findings. As indicated in lines 102 and 119, we added the target retrieval database and made the required adjustments in the body section.

**8. “The study has no restrictions on sex, race, duration of diabetes, or severity of diabetes among the participants” But is the severity of diabetes going to be controlled in some way?**

**Ans**：Thank you for your beneficial suggestions; we also considered patient safety when preparing the manuscript. As a result, we required that relevant disorders be diagnosed by professional physicians or competent healthcare institutions, and that patients receive counsel and guidance from professional physicians throughout the trial. At the same time, because this meta-analysis is a secondary review of the original study's data, it does not raise ethical or patient safety concerns. We did not limit the severity of diabetes in order to include as many relevant research as possible. If the data allow, we will undertake subgroup analyses based on diabetes severity to investigate the effect of diabetes severity on the connection between hypoglycemia and cognitive impairment.

**9. “…likelihood of cognitive dysfunction will be included”. Is there a way to determine if there is cognitive disorder? It is important to establish this a priori before the study.**

**Ans**: This is a critical and serious issue. Before creating this meta-analysis protocol, we conducted a pre-search of individual databases, and the majority of studies investigating the association between hypoglycemia and cognitive impairment performed cognitive function tests before individuals were enrolled. Subjects with impaired cognition were screened out because they did not match the inclusion criteria for these investigations since hypoglycemia was specified as an exposure factor and cognitive impairment was set as an outcome measure. This study was a meta-analysis of the findings from these studies, and cognitive impairment should be mentioned as an outcome indicator and its diagnostic criteria in the inclusion criteria. This was an oversight in the manuscript's preparation, which is now detailed in the inclusion criteria you requested, as indicated in lines 126-131.

**10. Inclusion criteria – “Studies not published in Chinese or English” and “Participants with gestational diabetes”. Exclusion criteria are not the opposite of inclusion criteria. Exclusion criteria are those that exclude participants or studies from inclusion even though they meet the inclusion criteria.**

**Ans**：Thank you for the suggestions. We removed the above two improper exclusion criteria based on your feedback.

**11. Search strategy – I suggest this section be placed before the eligibility criteria.**

**Ans**：Thanks for your suggestion, we have adjusted the order of the relevant content according to your suggestion, see lines 100 and 116 for details.

**12. Data extraction – “the primary author” ¿first author? “period of publication” ¿year of publication? “type of diabetes mellitus” Isn't it just type 2 diabetes mellitus? “type of cognitive impairment” The type of cognitive dysfunction that can be included needs to be defined beforehand.**

**Ans**：Thank you for your advice. We regret for the manuscript's confusing and erroneous description, and we have edited, eliminated, and replaced the appropriate text based on your suggestions. For more information, see lines 150-160.

**13. “Evaluation of literature quality (publication bias)” – First, how many authors did the publication bias assessment? Secondly, I do not recommend putting publication bias. Publication bias generally refers to the Egger test and the funnel plot for meta-analysis.**

**Ans**: Thank you for your advice. As per your suggestion, we have clarified in the publication that the assessment of the quality of the literature was conducted by two investigators, and any disagreements were resolved by a third investigator. We have removed the inappropriate phrase "publication bias" according to your suggestion. See lines 161-164 for details.

**14. Data synthesis – First, the authors should rate the heterogeneity, for example, as not important, moderate, substantial, and considerable. Second, the p-value of heterogeneity should be assessed. Third, why did you choose the value of 30% or more to do a random-effects meta-analysis? Why not 50%? Or why not if p is < 0.05? I suggest doing the random effects meta-analysis, studies with some heterogeneity in the population and its characteristics will be included, and fixed effects meta-analyses usually give narrower confidence intervals, which can be misleading in some contexts.**

**Ans**：Thank you for your valuable suggestions, which helped us clarify the heterogeneity rating. Initially, I^2^≥30% was chosen for random effect model analysis in order to estimate the effect size more conservatively, however after reviewing your idea, we believe that the previous consideration is unnecessary and erroneous. From this point on, we use the random effects model when either P < 0.1 or I^2^ ≥ 50%. Details can be found on lines 181-186.

**15. Publication bias – And what p-value is used as a threshold to consider that there is publication bias?**

**Ans**: Thanks for your suggestion, we have clarified the threshold of P value in the main text, when a P value < 0.05 is considered to have significant publication bias. See lines 175-186 for details.

**16. Grading the quality of evidence – It is proposed to explain it a little more (not much, but a little more, yes)**

**Ans**：Thanks for your guidance, we have elaborated the grading of the quality of evidence. See lines 207-212 for details.

**Response to Reviewer 4's Comments**

Firstly, we would like to express our sincere thanks for your constructive comments and positive response. The revised version of the manuscript has been significantly modified based on your comments. The revised parts are marked in red font. We hope you are satisfied with our revisions.

**1.I suggest including the specific study design (systematic review and meta-analysis protocol).**

**Ans**：We have added specific study designs to the abstract section as suggested by you. See line 23 for details.

**2. I recommend including both that this protocol will be adhered to the Preferred Reporting Items for Systematic Reviews and Meta-Analyses** **Protocols and the meta-analysis will be guided by the Cochrane Collaboration Handbook recommendations.**

**Ans**: We appreciate your insightful counsel, which we will take into consideration as we adhere to the Cochrane Collaboration Handbook recommendations and the preferred reporting items for the Systematic Reviews and Meta-analyses Protocols. We provide further details on this in the section on Methods and Materials, and in the Supplementary material, we include the updated PRISMA-P checklist. For details, see lines 92–99.

**3. Line 28: I suggest specifying the way in which the inconsistences will be solved.**

**Ans**: We appreciate your insightful counsel, and as per it, we have clarified that any discrepancies in the literature screening procedure were addressed by a different researcher. See line 33.

**4.Objective: The objective of this manuscript is not to explore the cumulative effect of the frequency of hypoglycemia events on cognitive function, but to establish the methodological structure (protocol) in order to explore, through systematic review and meta-analysis, the cumulative effect of the frequency of hypoglycemia events on cognitive function. Reconsider.**

**Ans**: Thank you for your constructive suggestions. We have revised the inappropriate expression according to your suggestions. See lines 86-89 for details.

**5.Line 89. The systematic review should also be adhered to the Meta-analysis Of Observational Studies in Epidemiology (MOOSE) statement.**

**Ans**: Thank you for your valuable suggestions. We have modified the relevant content according to your suggestions, which makes our scheme look more rigorous. See lines 96-97 for details.

**6. Line 96. Could the author please state the reason for the exclusion of experimental studies?**

**Ans**: Thank you for your question. First of all, this study aims to explore the relationship between hypoglycemic events (exposure factors) and cognitive impairment (outcome measures) in the natural state, and does not involve exploring the role of artificial interventions such as treatment measures. Second, hypoglycemic events are highly harmful to the human body and have unpredictable characteristics, so it is almost impossible to recruit subjects with stable hypoglycemia for experimental research due to ethical considerations. The current experimental studies on the relationship between hypoglycemic events and cognitive impairment are almost based on the construction of hypoglycemic animal models. Finally, this meta-analysis needs to use data such as odds ratio (OR), risk ratio (RR), hazard ratio (HR), and 95% confidence intervals (CI), which are not available in experimental studies. Therefore, only observational studies were included in this study.

**7.Line 156: It is suggested to indicate the range of heterogeneity based on I2 (not important, moderate, substantial, and considerable). Will the authors consider the corresponding p-value?**

**Ans**： Thank you for your proposal. We used the range of I^2^ values to grade the heterogeneity in accordance with your suggestion and the Cochrane Handbook. We also took into consideration that a P value < 0.1 was deemed to be significant heterogeneity. We have meticulously edited and illustrated the primary content. For more information, see lines 181–186.

**8.** **Line 190: The authors state that “This investigation seeks to explore the connection between hypoglycemic events and cognitive impairment”. However, they pretend to “clarify the precise association between hyperglycemia and different types of cognitive dysfunction”. Is this correct?**

**Ans：**Thank you for your careful review and finding this spelling error. We are very sorry for our carelessness. According to your suggestion, we have modified the text. See line 227 for details.
