# Supplementary material for: Association of hypoglycemic events with cognitive impairment in patients with type 2 diabetes mellitus: Protocol for a dose-response meta-analysis
Source: PLoS One. 2024 Feb 2;19(2):e0296662. doi: 10.1371/journal.pone.0296662 (PMC10836671; doi:10.1371/journal.pone.0296662)
Supplement: Attachment — Submitted filename: Response to Reviewers.docx [file pone.0296662.s006.docx]

**Response to Reviewers “Association of hypoglycemic events with cognitive impairment in patients with type 2 diabetes mellitus: Protocol for a dose-response meta-analysis”**

**Min Ye, Ai Hong Yuan, Qi Qi Yang, Qun Wei Li, Fei Yue Li, Yan Wei**

**Date	November 2023**

Dear Editor,

We would like to express our sincere thanks for giving us the valuable opportunity to revise our manuscript. Meanwhile, thanks to four reviewers for their careful and patient reviews.

The revised version of our manuscript has been modified based on their helpful comments. All the revisions to the manuscript are marked in red font. In the following correspondence, we describe the changes made in this version. We hope that now the paper is ready for publication.

Looking forward to hearing your response soon.

Thank you and best regards.

Yours sincerely,

Min Ye

Corresponding author:

Name: Ai Hong Yuan

E-mail: 490603279@qq.com; yuanah2023@163.com.

**Response to journal's requirements**

**1. Please review your reference list to ensure that it is complete and correct. If you have cited papers that have been retracted, please include the rationale for doing so in the manuscript text, or remove these references and replace them with relevant current references. Any changes to the reference list should be mentioned in the rebuttal letter that accompanies your revised manuscript. If you need to cite a retracted article, indicate the article’s retracted status in the References list and also include a citation and full reference for the retraction notice.**

**Ans**: I appreciate your kind reminder. We have conducted a thorough investigation of each reference once more by your recommendation. To standardize the reference format, all references were downloaded from PubMed and entered into the Endnote program. There is no issue number for references 7, 12, 13, 14, 39, and 43, and there is no digital object identifier number for reference 47 since it is not included in the original journal. Simultaneously, we examined all the references and were happy to see that none of the withdrawn publications had been cited. Once again, I appreciate your thorough review.

**Response to Reviewer 3's Comments**

Firstly, we would like to express our sincere thanks for your constructive comments and positive response. The revised version of the manuscript has been significantly modified based on your comments. The revised parts are marked in red font. We hope you are satisfied with our revisions.

**1.** **“There will be four categories for heterogeneity: not important (0%-40%), moderate (30%-60%), substantial (50%-90%) and considerable (75%-90%). The fixed effect model will be applied for combined analysis if the I^2^ value is less than 50%. A random effect model will be applied when P < 0.1 or I^2^ > 50%[32, 33]. Subgroup analysis and other techniques will be used to investigate sources of clinical or methodological heterogeneity when I^2^ > 75%.” - I suggest that considerable is 75-100%. I also suggest including subgroup studies even if there is no detected statistically significant heterogeneity.**

**Ans**: Regards for your insightful recommendations. About the I^2^ value classification, while there are currently a variety of versions, following a collaborative discussion among all authors, we consider your recommendations to be more feasible and scientifically sound, so we modify the considerable heterogeneity to 75–100% by your recommendations. We made adjustments for assertions that were not accurate. That is, regardless of whether statistically significant heterogeneity is found, subgroup analysis and other workable analytical techniques will be used to investigate clinical heterogeneity if there is enough literature. Details are shown in lines 182-187.

**2. Publication bias – Why is p < 0.10 not used to assume** **publication bias? It is the most commonly used value.**

**Ans**: We appreciate you informing us that two types of publication bias P-values are currently in use: 0.05 and 0.1. Since 0.1 is more rigorous and widely used, we have adjusted the P-value to 0.1 per your recommendation. For more information, see lines 206-207.

**Response to Reviewer 4's Comments**

Firstly, we would like to express our sincere thanks for your constructive comments and positive response. The revised version of the manuscript has been significantly modified based on your comments. The revised parts are marked in red font. We hope you are satisfied with our revisions.

**1. Line 85: I propose to rephrase the objective. I suggest the following: "This manuscript aims to establish a methodological structure (protocol) to explore the cumulative effect of frequency of hypoglycemic events on cognitive function and further explore the dose-response relationship through systematic review and meta-analysis, which aims to update the association between hypoglycemia and cognitive impairment by including the latest relevant studies.".**

**Ans**: We appreciate your patient advice, and we have adapted the research purpose in light of your recommendations, which improves the overall quality and scientific appearance of our program. For information, see lines 85–89.

**2. Could the authors please cite the MOOSE statement?**

**Ans**: We appreciate you reminding us and apologize for this error. As per your recommendation, we have now included the pertinent references of the MOOSE statement. For information, see lines 96–97 and 356–359.
