# Supplementary material for: Association of hypoglycemic events with cognitive impairment in patients with type 2 diabetes mellitus: Protocol for a dose-response meta-analysis
Source: PLoS One. 2024 Feb 2;19(2):e0296662. doi: 10.1371/journal.pone.0296662 (PMC10836671; doi:10.1371/journal.pone.0296662)
Supplement: Attachment — Submitted filename: Response to Reviewers.docx [file pone.0296662.s007.docx]

**Response to Reviewers “Association of hypoglycemic events with cognitive impairment in patients with type 2 diabetes mellitus: Protocol for a dose-response meta-analysis”**

**Min Ye, Ai Hong Yuan, Qi Qi Yang, Qun Wei Li, Fei Yue Li, Yan Wei**

**Date	December 2023**

Dear Editor,

We would like to express our sincere thanks for your helpful comments and giving us the valuable opportunity to revise our manuscript. These comments are all valuable and very helpful for revising and improving our manuscript, as well as the important guiding significance to our study. We have studied the comments carefully and have made corrections, which we hope to meet with approval. Meanwhile, thanks to two reviewers for their careful and patient reviews.

The revised version of our manuscript has been modified based on those helpful comments. All the revisions to the manuscript are marked in red font. In the following correspondence, we describe the changes made in this version. Please contact us if there is anything that has not been completely modified or does not meet the requirements.

Looking forward to hearing your response soon.

Thank you and best regards.

Yours sincerely,

Min Ye

Corresponding author:

Name: Ai Hong Yuan

E-mail: 490603279@qq.com; yuanah2023@163.com.

**Response to journal's requirements**

**1. Please review your reference list to ensure that it is complete and correct. If you have cited papers that have been retracted, please include the rationale for doing so in the manuscript text, or remove these references and replace them with relevant current references. Any changes to the reference list should be mentioned in the rebuttal letter that accompanies your revised manuscript. If you need to cite a retracted article, indicate the article’s retracted status in the References list and also include a citation and full reference for the retraction notice.**

**Ans**: I appreciate your kind reminder. We have conducted a thorough investigation of each reference once more by your recommendation. To standardize the reference format, all references were downloaded from PubMed and entered into the Endnote program. There is no issue number for references 7, 12, 13, 14, 38, 40, and 44, and there is no digital object identifier number for reference 48 since it is not included in the original journal. Simultaneously, we examined all the references and were happy to see that none of the withdrawn publications had been cited. Once again, I appreciate your thorough review.

**Response to Reviewer 3's Comments**

Firstly, we would like to express our sincere thanks for your constructive comments and positive response. The revised version of the manuscript has been significantly modified based on your comments. The revised parts are marked in red font. We hope you are satisfied with our revisions.

**1.** **“There will be four categories for heterogeneity: not important (0%-40%), moderate (30%-60%), substantial (50%-90%) and considerable (75%-90%). The fixed effect model will be applied for combined analysis if the I^2^ value is less than 50%. A random effect model will be applied when P < 0.1 or I^2^ > 50%[32, 33]. Subgroup analysis and other techniques will be used to investigate sources of clinical or methodological heterogeneity when I^2^ > 75%.” - I suggest that considerable is 75-100%. I also suggest including subgroup studies even if there is no detected statistically significant heterogeneity.**

**Ans**: Regards for your insightful recommendations. About the I^2^ value classification, while there are currently a variety of versions, following a collaborative discussion among all authors, we consider your recommendations to be more feasible and scientifically sound, so we modify the considerable heterogeneity to 75–100% by your recommendations. We made adjustments for assertions that were not accurate. That is, regardless of whether statistically significant heterogeneity is found, subgroup analysis and other workable analytical techniques will be used to investigate clinical heterogeneity if there is enough literature. Details are shown in lines 192-197.

**2. Publication bias – Why is p < 0.10 not used to assume** **publication bias? It is the most commonly used value.**

**Ans**: We appreciate your suggestion, but regrettably, we would still like to use *P* < 0.05 as the standard for determining publication bias for the reasons listed below.

1. After searching across several databases for relevant meta-analyses, we discovered that the most widely used value for assessing publication bias remains 0.05, with only a tiny number of studies using 0.10.

2. The commonly used threshold for statistical significance in scientific research is *P* < 0.05, which means that the observed results are unlikely to have occurred by chance alone. However, the use of *P* < 0.10 to assume publication bias is not a standard practice. The threshold of *P* < 0.05 is widely accepted in the scientific community and is considered a standard level of significance.

3. The rationale behind using a stricter threshold for publication bias assessment is to reduce the risk of false positives or Type I errors. Setting a more lenient threshold, such as p < 0.10, increases the likelihood of incorrectly concluding that there is publication bias when it may not be present.

**Response to Reviewer 4's Comments**

Firstly, we would like to express our sincere thanks for your constructive comments and positive response. The revised version of the manuscript has been significantly modified based on your comments. The revised parts are marked in red font. We hope you are satisfied with our revisions.

**1. Line 85: I propose to rephrase the objective. I suggest the following: "This manuscript aims to establish a methodological structure (protocol) to explore the cumulative effect of frequency of hypoglycemic events on cognitive function and further explore the dose-response relationship through systematic review and meta-analysis, which aims to update the association between hypoglycemia and cognitive impairment by including the latest relevant studies.".**

**Ans**: We appreciate your patient advice, and we have adapted the research purpose in light of your recommendations, which improves the overall quality and scientific appearance of our program. For information, see lines 85–89.

**2. Could the authors please cite the MOOSE statement?**

**Ans**: We appreciate you reminding us and apologize for this error. As per your recommendation, we have now included the pertinent references of the MOOSE statement. For information, see lines 96–97 and 393–396.

**Response to Editor's Comments**

**1. As an editor, suggest it to keep statistical significance in scientific research is p < 0.05. I have given my details below.** **Moreover, Please explain in detail the Publication bias. The current has no justification and explanation.**

**Ans**: Dear editor, thank you for your advice. After consultation with all the authors, we decided to adopt your suggestion and retain a *P* value of less than 0.05 as the measure of publication bias. For the following reasons:

(1) Within the scientific world, the threshold of *P*<0.05 is commonly acknowledged as the conventional level of significance. We examined meta-analyses from several databases, and it was found that the majority of writers continued to use *P*< 0.05 as the standard for evaluating publication bias.

(2) The reviewers recommend a looser criterion of *P* < 0.10, which will boost the sensitivity of the detection of publication bias. However, it may also lead to an incorrect conclusion that publication bias may be present in the findings when it might not be. As a result, we eventually chose to keep *P* < 0.05 as our publishing bias metric.

(3) Furthermore, we have included the potential for publication bias in our study protocol in the text. There was an explanation of pertinent potential publishing bias in detail. Refer to lines 214–222.

**2. Please Revisit the exclusion criteria. I am giving some suggestion. It is not mandatory to include all suggestion. yet your study exclusion criteria is important.**

**Ans**: Thank you for your valuable advice, which is very important to us. We have appropriately supplemented the exclusion criteria according to your suggestion, which makes our study protocol more rigorous and complete. See lines 137-149 for details. However, we did not use all of these recommendations as exclusion criteria for the following reasons:

(1) The inclusion criteria refer to the basic conditions for enrollment, while the exclusion criteria should be other special conditions that do not meet the requirements of the trial based on the inclusion criteria, rather than the opposite of the inclusion criteria. Therefore, suggestions about the opposite of the inclusion criteria, such as “studies that focus exclusively on populations other than patients with type 2 diabetes mellitus”, “studies with mixed populations where data on patients with type 2 diabetes cannot be extracted separately”, “studies that do not report hypoglycemic events”, “studies that do not report cognitive impairment as an outcome”, “studies that use different definitions or measurements of cognitive impairment”, “studies that are not primary research articles (e.g., reviews, editorials, commentaries)”, and “studies not reporting relevant effect measures” will not be included in the exclusion criteria, because these studies would not have been considered for inclusion in the first place.

(2) Furthermore, as stated in the inclusion criteria, original studies can be included as long as they provide pertinent data for the dose-response relationship, and the dose-response relationship was established by a meta-analysis synthesizing data from multiple original studies. Therefore, "observational studies lacking a clear dose-response relationship" will not be considered an exclusion criterion. All initial research doesn't need to have been done with dose-response relationship calculations.

(3) The results of the literature pre-search indicate that there hasn't been any notable advancement in our understanding of diabetes-related cognitive impairment, and the inclusion criteria specify the methodologies used to measure cognitive impairment. As there is nearly no earlier research remaining after rigorous screening in accordance with the inclusion criteria, it is not required to consider omitting earlier research at this time.

**3. Please give two heading study strength and study limitation and explain in depth.**

**Ans**: We appreciate your proposal, and as stated in lines 290 to 305 of the text, we added both the strengths and limitations of this study protocol.

4. **The rationale to use Grading the quality of evidence is not clear. Explain in depth. It is suggestion compare GRADE with AMSTAR 2 (A MeaSurement Tool to Assess systematic Reviews), AHRQ (Agency for Healthcare Research and Quality) Methods Guide and ROBIS (Risk of Bias in Systematic Reviews) Tool.**

**Ans**: We appreciate your constructive guidance. We examined four different tools (GRADE, AMSTAR 2, AHRQ, and ROBIS) based on your recommendations. In the end, we chose to evaluate the quality of the evidence using two tools: GRADE and ROBIS (see lines 36–37 and 228–236). The following are the reasons.

(1) AMSTAR 2 applies to systematic reviews of randomized controlled trials, non-randomized intervention studies, or both. This study protocol will include observational studies to explore the relationship between exposure factors and outcome measures and does not involve intervention studies, so AMSTAR 2 is not suitable for this study protocol.

(2) AHRQ Methods Guide recommends the criteria for quality assessment of observational studies, which assesses the risk of bias from five domains: selection bias, performance bias, follow-up bias, measurement bias, and reporting bias. It can assess the risk of bias in different types of original studies included in the same systematic review but does not give recommendations on how to determine the overall risk of bias in the review. As for the original study, the NOS scale, which is more commonly used and convenient, is intended to be used in this study. The NOS scale is a good combination of the reality of case-control studies and cohort studies, which is more in line with this study protocol. Therefore, the AHRQ Methods Guide will not be used in this study.

(3) Unlike the AMSTAR 2 tool, which concentrates on evaluating the methodological quality of systematic reviews, ROBIS is committed to evaluating the risk of bias in the production of systematic reviews. ROBIS is suitable for our research protocol because it may be used for systematic reviews of many study types, such as prognostic, etiologic, interventional, and diagnostic test reviews.

(4) The GRADE criteria explicitly define the quality of evidence and the strength of recommendations, completely abandoning the practice of grading evidence based on the type of study design and instead considering the type of study design, methodological quality, consistency of results, and directness of evidence.

(5) The methodological quality of systematic reviews reflects only part of the risk of bias, so it seems more reasonable for reviewers to evaluate the quality of systematic reviews using different tools at the same time, complementing each other. For the final evaluation of the quality of the evidence, we therefore chose to employ the GRADE criteria and ROBIS, taking into account the specific circumstances of the study protocol.
